# Shift in *Staphylococcus aureus* Clone Linked to an Infected Tattoo

**DOI:** 10.3201/eid1209.051634

**Published:** 2006-09

**Authors:** Mary E. Stemper, Jennifer M. Brady, Salah S. Qutaishat, Gwen Borlaug, James Reed, Kurt D. Reed, Sanjay K. Shukla

**Affiliations:** * Marshfield Laboratories, Marshfield, Wisconsin, USA;; † Marshfield Clinic Research Foundation, Marshfield, Wisconsin, USA;; ‡ Saint Joseph's Hospital, Marshfield, Wisconsin, USA;; § Bureau of Communicable Diseases, Madison, Wisconsin, USA;; ¶ Oxford Correctional Facility, Oxford, Wisconsin, USA

**Keywords:** Community-associated, correctional facility, MRSA, Staphylococcus aureus, methicillin resistance, outbreak, Panton-Valentine leukocidin, USA300 clone, skin and soft tissue infections, tattoo, toxin, virulence, dispatch

## Abstract

A retrospective investigation of skin and soft tissue infections caused by community-associated methicillin-resistant *Staphylococcus aureus* (MRSA) strains among inmates in a Wisconsin correctional facility suggested a shift in MRSA genotype. Case timeline indicated a displacement of USA400 clone by USA300 clone. The USA300 index case was associated with an infected new tattoo.

Community-associated methicillin-resistant *Staphylococcus aureus* (CA-MRSA) is phenotypically and genotypically different from healthcare-associated MRSA (HA-MRSA). Also, risk factors for acquiring CA-MRSA infections differ from those for acquiring HA-MRSA and include crowding, close contact, lack of cleanliness, compromised skin, and contaminated fomites. These risk factors have enabled CA-MRSA to infect persons who meet >1 of these criteria.

In the 1990s, outbreaks of CA-MRSA–related infections occurred primarily in certain groups, such as Native Americans, before disseminating into the general population (*1**–**5*). More recently, CA-MRSA outbreaks were seen in unsuspected groups such as military personnel (*6**,**7*), athletes (*8**,**9*), and inmates at large correctional facilities (*10**,**11*). Molecular typing of strains from these recent outbreaks showed that most differed from the predominant clone of the 1990s and belonged to a new CA-MRSA clone, USA300 (*9*). In this retrospective study, we report an outbreak of CA-MRSA–associated skin and soft tissue infections (SSTIs) among inmates of a medium-size correctional facility in Wisconsin. This outbreak was caused by USA400 strains but appeared to be displaced by USA300 clonal group after it was introduced into the facility.

## The Study

**Figure 1:**
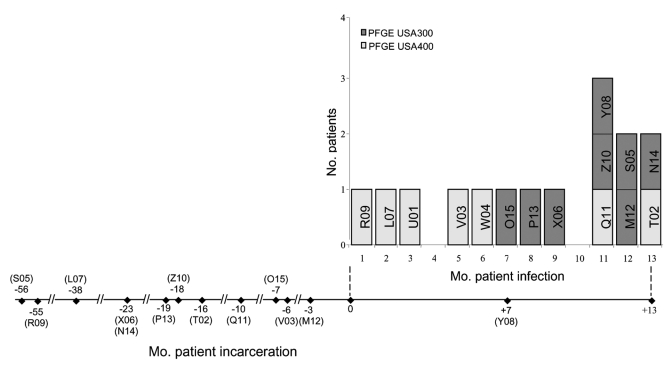
Timeline of incarceration and isolation of methicillin-resistant *Staphylococcus aureus* isolates from different patients. Top panel: baseline shows months in which a particular isolate was recovered and patient was identified as infected; y-axis shows number of patients in each clonal group per month during the outbreak period. Bottom panel: horizontal line shows duration in which patients were incarcerated in relation to the outbreak period. Month 0 and month numbers with – and + symbols represent the respective months of incarceration before and after onset of the outbreak, respectively. Codes below months represent patients.

This study was approved by the Institutional Review Board of Marshfield Clinic and Marshfield Clinic Research Foundation. Fifteen MRSA isolates were recovered from 15 patients in a correctional facility over a 13-month period (Figure 1). These isolates were recovered from SSTI wound samples submitted to Marshfield Laboratories. The patients were housed in 7 of 10 units with a common recreation yard at a 1,200-inmate facility in Wisconsin from May 2002 to May 2003. Infections with MRSA were rare in this facility; the last reported case of MRSA was 16 months ago. Because of increased number of SSTIs during this period, the Wisconsin Division of Public Health initiated an investigation to determine whether these strains were epidemiologically related.

All strains were typed by pulsed-field gel electrophoresis (PFGE) and staphylococcal cassette chromosome *mec* (SCC*mec*) and tested for virulence genes. Only the first isolate of each PFGE-based clonal group and 1 additional isolate from the same clone were analyzed with *spa* and multilocus sequence typing.

**Figure 2:**
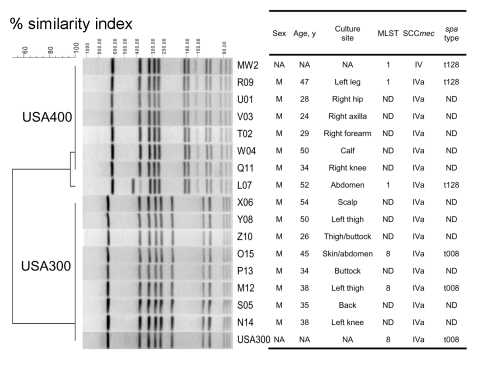
Pulsed-field gel electrophoresis (PFGE)–based dendrogram of methicillin-resistant *Staphylococcus aureus* strains isolated during the outbreak. A genetic similarity index scale is shown above the dendrogram. Strain numbers, clone identification, site of infection, and demographic information are included along each PFGE lane. MLST, multilocus sequence typing; SCC*mec*, staphylococcal cassette chromosome *mec*; NA, not available; ND, not determined.

All 15 patients were men (average age 39 years) and had SSTIs at various body sites (Figure 2). All patients, except for inmate Y08, who entered the facility in the seventh month of the outbreak (Figure 1), were incarcerated for 3 to 56 months before the outbreak. Dates of incarceration for 2 inmates could not be determined. All 15 isolates were resistant to β-lactams but sensitive to ciprofloxacin, gentamicin, rifampicin, tetracycline, trimethoprim-sulfamethoxazole, and vancomycin.

PFGE analysis grouped these isolates into 2 clonal groups, USA400 (n = 7, 47%) and USA300 (n = 8, 53%) (Figure 2). The first isolate and a randomly selected second isolate of USA300 and USA400 clonal groups were determined to be sequence type (ST) 8 and ST1, respectively. The representative USA300 strains were *spa* type t008 (YHGFMBQBLO), and USA400 strains were *spa* type t128 (UJJFKBPE). All 15 strains in both clonal groups were type IVa SCC*mec* and positive for virulence factor Panton-Valentine leukocidin (PVL) genes (*lukSF*-*PV*) and staphylococcal enterotoxin gene *sek*. Isolates of USA400 were also positive for *sea*, *sec*, *seh*, *sel*, and *fnbA*. USA300 strains were positive for *fnbA* and *fnbB*.

Both PFGE profiles (Figure 2) of isolates of USA400 clone were previously observed in Native American communities in Wisconsin throughout the 1990s (*4*). However, ethnicity of the patients in the current study was not determined. PFGE profiles of USA300 strains were indistinguishable from USA300–0114 type strain (*9*). Like USA400 strains, USA300 strains were sensitive to many classes of antimicrobial drugs. However, unlike type 0014 strain, USA300 strains in this study were sensitive to tetracycline. All 8 strains in the USA300 clonal group were resistant to erythromycin but lacked inducible clindamycin resistance by the D-test (data not shown), whereas only 3 (43%) USA400 isolates were resistant to erythromycin and showed inducible clindamycin resistance.

After the outbreak investigation, the facility instituted specific infection control measures for inmates and staff members. Measures included promoting frequent hand washing and improving sanitation of laundry, linens, showers, bathrooms, and equipment in the recreation yard. Inmates were educated about personal hygiene and consequences of sharing needles and other sharp objects. Subsequently, the number of MRSA cases in this facility decreased substantially from 1.25 cases per month during the study period to 0.67 cases per month over the next 6 months.

## Conclusions

Molecular typing data for most reported CA-MRSA outbreaks in athletes and prisoners since 2000 showed that these strains belonged to the USA300 clonal group (*9*). This clone has also been reported in the general community in Michigan, predominantly among young African Americans with SSTIs (*12*). Recent reports also indicate that infections with USA300 strains are emerging in neonatal and pediatric groups (*13**,**14*). Therefore, this new CA-MRSA clone is not restricted to groups initially reported (*6**–**11*) but has reached the community at large (*12**–**14*).

When and how USA300 clone became established in this Wisconsin correctional facility were not clear. Since interstate transfer of inmates between correctional facilities is common, we speculate that the USA300 clone might have been introduced from such a transfer. The suspected carrier of USA300 clone in this facility could be a colonized inmate (Y08), who was incarcerated in the same month in which the first USA300 strain was identified. The initial case (O15) of the USA300 clone was in an inmate with an infection of a new abdominal tattoo acquired in this facility. The transferred inmate (Y08) was eventually identified as infected with an identical strain in the eleventh month of the outbreak. We speculate that USA300 probably spread among the inmates who were in close contact and shared fomites extensively. Sharing needles and tattoo paraphernalia is common in many correctional facilities. Irrespective of the mechanism of introduction, subsequent SSTIs reported from this facility were mainly due to the newly introduced clone.

USA300 appears to have become the new dominant CA-MRSA clone in a Wisconsin correctional facility, similar to what has occurred in other facilities in the United States. This clonal displacement could be due to better fitness of the USA300 clone than the USA400 clone in vulnerable groups who frequently have >1 risk factor. However, fitness factors that impart advantage to USA300 strains are not clearly identified. Tenover et al. compared genomes of several USA300 strains with USA400 and USA500 and USA100 strains (the last 2 are of healthcare-associated lineages) (*15*). They reported that USA300 strains have several unique sequences in pathogenicity islands such as phi PVL, phi N315, and SaPIn2, in addition to genes encoding several fibronectin-binding proteins such as *fnbA*, *fnbB*, and *ebh*.

Our limited virulence data for USA300 strains in this study showed that they were positive for *fnaA* and *fnaB*, but lacked enterotoxin genes *sea*, *sec*, *she*, and *sel*, some of which are frequently present in the USA400 strains. Genome sequence data from multidrug-resistant USA300 strain FPR3757 showed that it has a novel mobile genetic element that contains genes for enzymes of the arginine deiminase pathway and an oligopeptide permease system (*16*). It is speculated that the arginine catabolic mobile element, which is common in *S*. *epidermidis* but not in *S*. *aureus*, probably offers a selective advantage and contributes toward enhanced growth and survival of USA300 on human skin (*16*).

Although risk factors such as close contact, crowded environment, contaminated fomites, lack of cleanliness, and most importantly, compromised skin barriers are crucial in transmitting CA-MRSA–related infections, the role of unknown genomic fitness or virulence factors of USA300 strains cannot be underestimated in its recent spread. Whether certain conditions besides those mentioned also favor establishment of one clone of CA-MRSA over another in the community settings is also not clear (*17*).

We document gradual clonal displacement of USA400 by USA300 in a Wisconsin correctional facility. Sharing of tattoo paraphernalia may be associated with the outbreak and could be considered a possible risk factor for spread of CA-MRSA.
